# The glycoproteomics of hawk and caiman tears

**DOI:** 10.1186/s12917-021-03088-1

**Published:** 2021-12-09

**Authors:** Ana Cláudia Raposo, Carlito Lebrilla, Ricardo Wagner Portela, Gege Xu, Arianne Pontes Oriá

**Affiliations:** 1 School of Veterinary Medicine, Federal University of Bahia, Brazil, Salvador, 40.110-060 Brazil; 2 Chemistry Department, Mass Spectrometry Facilities Campus, University of California, Davis, CA 95616–8585 USA; 3 Institute of Health Sciences, Federal University of Bahia, Brazil, Salvador, 40.110-100 Brazil

**Keywords:** ]Bird, Clinical biochemistry, Ocular surface, Reptile, Tear film

## Abstract

**Background:**

Glycoproteins are important tear components that participate in the stability of the ocular surface. However, the glycopeptides that are present in the tears of wild animals have not yet been described. This work aimed to describe the glycoproteomic profile of roadside hawk (*Rupornis magnirostris*) and caiman (*Caiman latirostris*) tears.

**Methods:**

Tears collected from 10 hawks and 70 caimans using Schirmer tear test strips were used in this study. The samples were submitted to trypsin digestion and separated using a reverse-phase column coupled to a mass spectrometer associated to a nanospray ionization source. The glycoproteins were categorized as: cellular components, biological processes and molecular function, according to the UniProt Knowledgebase.

**Results:**

As shown by the liquid chromatography–mass spectrometry, all glycopeptides found were classified as N-type. Of the 51 glycoproteins that were identified in the hawk tear film, the most abundant were ovotransferrin, globulins and complement system proteins. In the caiman tear film, 29 glycoproteins were identified. The most abundant caiman glycoproteins were uncharacterized proteins, ATPases, globulins and proteasome components. Ontological characterization revealed that the glycoproteins were extracellular, and the most identified molecular function was endopeptidase activity for both species.

**Conclusion:**

Glycoproteins are abundant in the tear film of the bird and reptile species studied herein, and all these molecules were shown to have N-type modifications. Location at the extracellular space and an endopeptidase inhibitor activity were the main cell component and molecular function for both species, respectively. These profiles showed differences when compared to human tears, are possibly linked to adaptive processes and can be the basis for further studies on the search of disease biomarkers.

**Supplementary Information:**

The online version contains supplementary material available at 10.1186/s12917-021-03088-1.

## Background

Glycosylation is associated with important post-translational modifications (PTMs) in eukaryotes and is involved in several processes, such as cell reorganization and immune response. PTMs are linked to the structural heterogeneity among vertebrates [1–3]. In ophthalmology, studies on glycosylation and glycoproteins have revealed important systemic and ophthalmic biomarkers of diseases [4], such as the association of an altered glycosylation with dry eye diseases and ocular rosacea [5–7].

The functions of the tear film include protection, nutrition, and maintenance of the ocular surface. The tear fluid is the interface between the ocular surface and the environment, and it is highly influenced by both [8–10]. The human tear film is composed of lipids, proteins, salts, water and mucins [8, 11]. In mammals, glycoproteins such as mucins participate in the anchoring of the tear film on the ocular surface and in the localized immune response [6–8]. Thus, a description of peptide-related glycosylation can be a key for the elucidation of tear-stabilization mechanisms under different environmental conditions.

The knowledge of tear film glycoprotein profiles has been enhanced by the introduction of methodologies such as mass spectrometry (MS) [3], which provides descriptions of peptides, glycosylation sites and interactions between oligosaccharides [12]. Several research groups have demonstrated that PTMs present in other fluids, such as blood serum, plasma [1, 2] and saliva [5], can act as biomarkers for the diagnosis and prevention of diseases [4]. Indeed, the wide diversity of PTMs poses a challenge to an overall functional description. Different methodologies, such as electrophoresis and MS, have shown a similar glycoprotein composition for tears of humans and other mammals, such as cows, sheep and camels [13]. In human tears, 43 N-linked glycoproteins have been described [12], and 10 different mucin types (O-linked) compose between 50–90% of the total tear mucin mass [6].

Vertebrates interact with the environment mainly through their sense of sight [14]. However, little is known about the tear film composition of birds and reptiles, which are exposed to intense air friction during flight and to dense and dirty water, respectively [10, 15, 16]. Previous studies showed differences between the tear protein profiles in birds, reptiles, and mammals [10, 17, 18]. Glycoproteins found in bird and reptile tear films may present a high biotechnological potential for the development of new ophthalmic drugs, such as lacrimomimetics and therapeuticals with immunological functions. The knowledge of the normal composition of this fluid can be the basis for further studies on the adaptation characteristics and differences among healthy and disease status. Therefore, the aim of this work was to describe the glycoproteins present in the tear film of birds and reptiles, using the roadside hawk (*Rupornis magnirostris*) and the broad-snouted caiman (*Caiman latirostris*) as respective models.

## Results

Samples were successfully obtained from 10 hawks and 70 caimans. One caiman was excluded from the study since it presented with a cataract in both eyes. Samples from two hawks were excluded because the strip fell out and was contaminated.

The liquid chromatography–mass spectrometry (LC–MS) identified 51 glycoproteins in the hawk tear film unit, all of them classified as N-type; 7 proteins had a carbon-like modification (C) in addition to a *N*-group binding (Asn-x-Ser/Thr), with N-acetylglucosamine being the most common disaccharide (Supplementary Table 1). In caiman tears, 29 glycoproteins were identified, with N-type modifications observed for all oligosaccharides. Compared to birds, the caiman tear film presented a higher number of fucose bonds, and glucose and N-acetylglucosamine were more abundant (Supplementary Table 2).

The glycoprotein ontological classification was similar to the one found in studies on human’s tear proteins, such that the research criterion used herein was set to the taxa Aves (class) and Alligatoridae (family) for hawk and caiman, respectively. If the identified proteins were not found at the UniProt Knowledgebase (UPKB), they were described as "not characterized”.

**Fig. 1:**
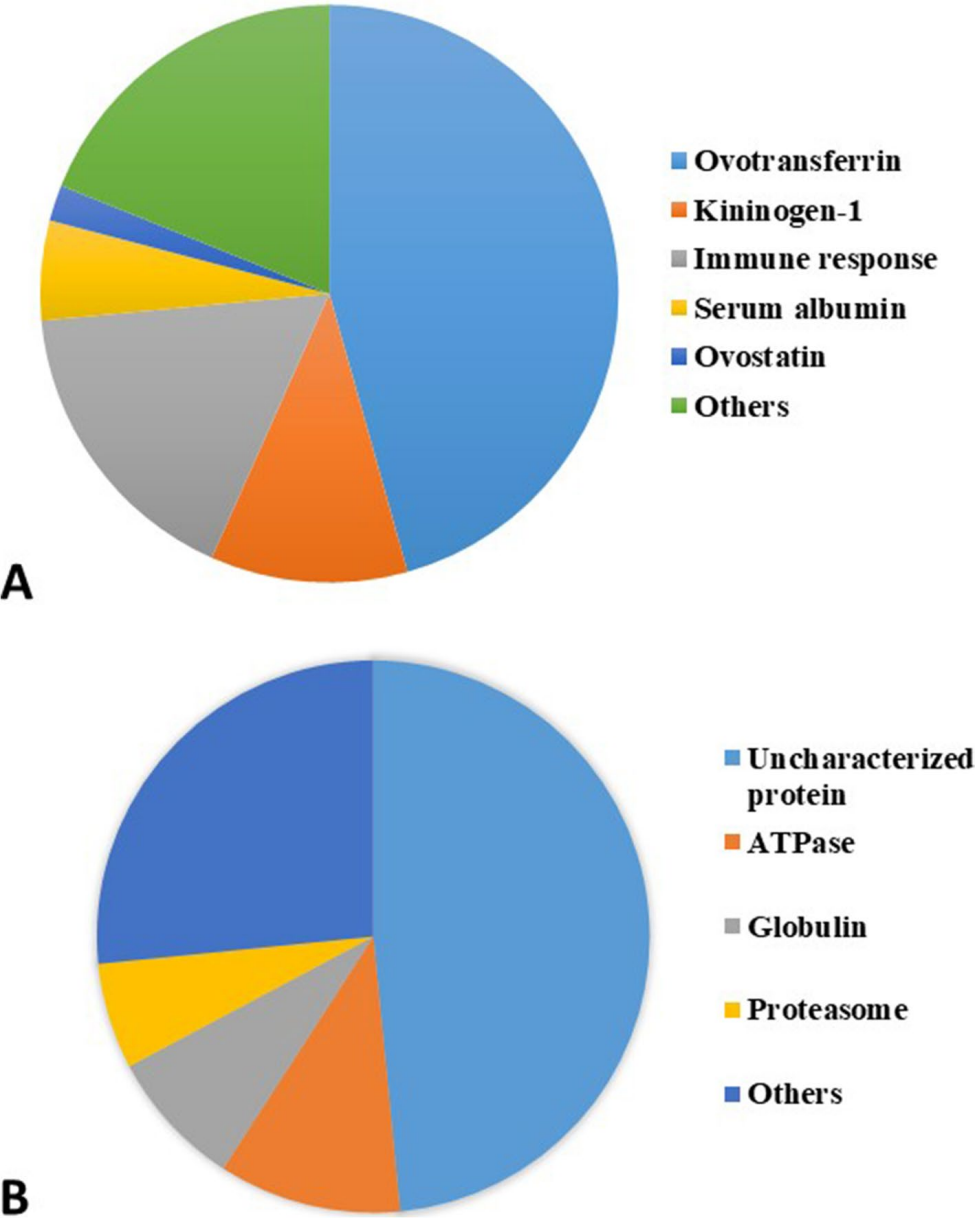
Distribution of protein abundance in *Rupornis magnirostris* (**A**) and *Caiman latirostris* (**B**) tear glycoproteins. Different shades of the same color do not correspond to proximity or similarity among proteins

The most abundant glycoproteins in the hawk tear film were ovotransferrin (45.6%), multiple glycoproteins classified into the immune response function category (16.8%), kininogen-1 (11.0%), serum albumin (5.6%) and ovostatin (2.0%). Other proteins occurred less frequently (<2% each), and together made up about 19% of the total protein abundance in the hawk tear film (Fig 1A). In the caiman tear film, we observed ten uncharacterized proteins (48.4%total), ATPases (10.7%), globulin (8.1%), proteasome components (6.2%), and less frequently other abundant proteins that together made up 26.6% of the sample (<2% each) (Fig 1B). Table 1 shows the glycoproteins with the highest abundance in hawk and caiman tear film when compared to the human tear film.

**Table 1:** Glycoproteins with the highest abundance in roadside hawk, broad-snouted caiman and human tears

Roadside hawk	Broad-snouted caiman	Human [12, 19]
Ovotransferrin	Uncharacterized proteins^a^	Lacritin
Alpha-2-macroglobulin-like 1	ATPase family AAA domain-containing protein 5	Galectin-3 binding protein
Kininogen-1	Alpha-2-macroglobulin-like protein 1	Clusterin
Serum albumin	Proteasome subunit alpha type	Lactoferrin
Ovostatin	Calumenin	Immunoglobulin isoforms

^a^Ten uncharacterized proteins were found in caiman tears

**Fig. 2:**
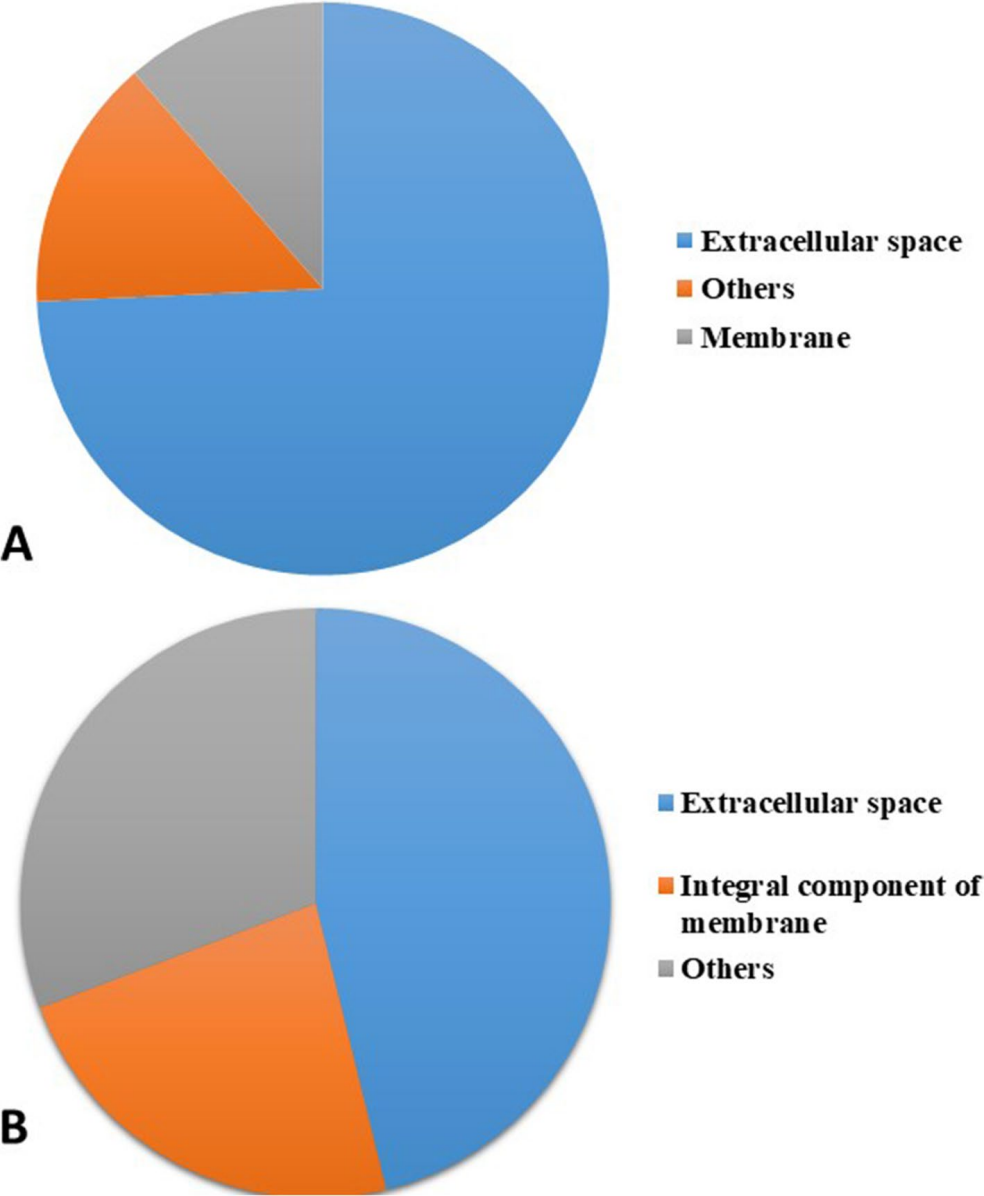
Frequency distribution of the cellular components identified in *Rupornis magnirostris* (**A**) and *Caiman latirostris* (**B**) tear glycoproteins. Different shades of the same color do not correspond to proximity or similarity among proteins

The glycopeptides that were included in the cell component category were classified into 34 and 14 different classifications within this specific category for hawk and caiman, respectively, following the UPKB database (Fig 2A and B). Within the 34 glycoproteins categorized as cell components for hawks, the more frequent classification was extracellular, and this same situation could be noted for caimans. Other classifications were, for example: the hawk tear glycoprotein actin was classified as cytoskeleton; for caiman, the glycoprotein proteasome was classified as cytoplasm. The most abundant glycoproteins in the hawk (ovotransferrin) and caiman tear film (uncharacterized protein) were also classified as extracellular. According to UPKB, the biological processes found for the hawk tear glycoproteins were blood coagulation, immune response, collagen organization, cell wall macromolecule catabolic process, glucose metabolic process, ion binding, establishment of Golgi localization, actin filament severing and regulation of cell proliferation. In caiman tears, the glycoprotein biological processes were complement activation, cytoskeletal anchoring at the nuclear membrane, ubiquitin-dependent protein catabolic process, homophilic cell adhesion via plasma membrane adhesion molecules, glycolytic process, regulation of transcription by RNA polymerase II and peptide crosslinking.

**Fig. 3:**
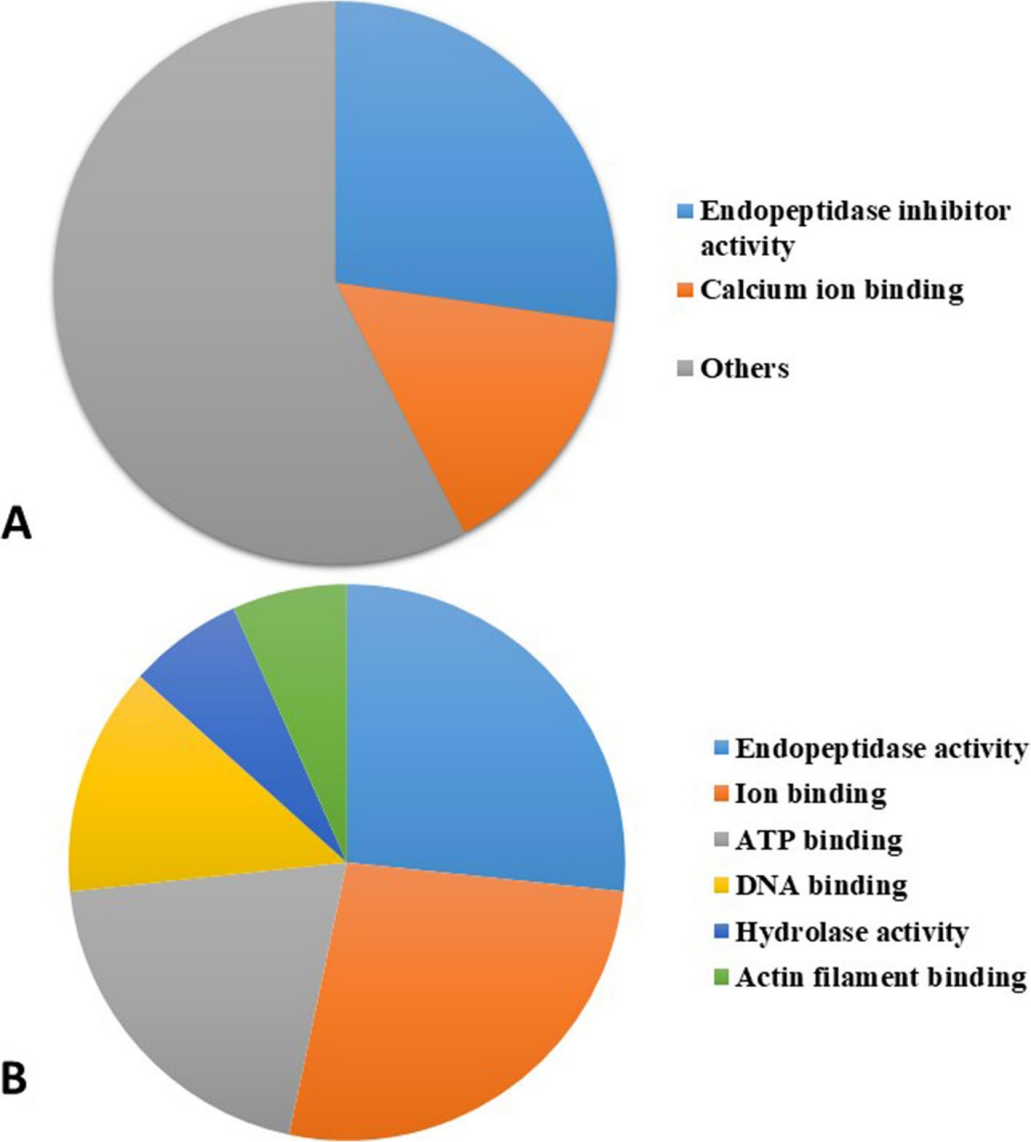
Frequency distribution of the molecular functions identified for *Rupornis magnirostris* (**A**) and *Caiman latirostris* (**B**) tear glycoproteins. Different shades of the same color do not correspond to proximity or similarity among proteins. The presented data are not necessarily correlated to the most abundant proteins.

According to UPKB, the molecular function category of hawk tears was more diverse than that of caiman tears, with 33 and 14 molecular functions, respectively. Endopeptidase inhibition and ion binding were the most frequent functions for both species’ tear glycoproteins (Fig 3A and B). The ovotransferrin molecular function, the most frequent one in hawk tears, was described as ion binding, whereas the uncharacterized proteins found in the caiman was characterized as having endopeptidase inhibitory activity. The molecular function glycoproteins with the highest abundance in hawk and caiman tear film have similarities and differences when compared to the human tear film (Table 2).

**Table 2:** Molecular function with the highest abundance in roadside hawk, broad-snouted caiman and human tears

Roadside hawk	Broad-snouted caiman	Human [12, 19]
Endopeptidase inhibitor activity	Endopeptidase inhibitor activity	Ion binding
Calcium ion binding	Ion binding	Transcytosis of soluble dimeric IgAs
Heparin binding	ATP binding	Enzyme activator activity
Polysaccharide binding	DNA binding	Modulation of lymphocyte proliferation
Phosphatase activity	Hydrolase activity	Cell adhesion molecule binding

## Discussion

Among the tear components, glycoproteins are potentially important biomarkers; in particular, N-linked glycocomponents have been widely studied in pathological conditions [4, 12]. Advances in the methodologies describing PTMs have led to the understanding of the different tear stabilization mechanisms, especially when it is exposed to extreme environmental conditions [5, 10, 12, 16]. Descriptions of wild animals’ tear components are scarce [10, 16, 17], and most of the currently available studies on this are have been conducted on humans [18]. A description of the tear glycoproteins from bird and reptile species can provide information that could explain how the tear film remains well preserved across orders even under challenging conditions. Our study represents the first description of non-mammalian tear glycoproteins.

Research reports on the proteomic profile of the human tear film have revealed from 490 to 1500 different proteins, using different MS methodologies [20–22], which demonstrates the complexity and heterogeneity of this fluid. In studies focused on glycocomponents, LC–MS presented high precision in the detection of biomarkers [23].

The tear film structural matrix of some reptiles, birds and mammals is similar to that described for the human tear film [16, 24] and, considering this similarity, the same methodology can be used. The UPKB was used to classify the glycopeptides since it has a more diverse genetic bank information, and it is possible to obtain the best protein characterization. The most appropriate situation would be a species-specific match for proteins classification; however, this fact was not possible due to the fact that the currently available genetic banks for animals are scarce and limited.

The number of N-linked glycoprotein types found in hawk tear film was close to that described for humans (51 in the hawk vs. 47 in humans [12]). In caimans, there were a higher number of fucosylations (a peptide linked to hexose deoxy sugar, with an important immunological function in humans [25]), possibly correlated with microbial defense mechanisms, as previously observed in human breast milk [25]. However, due to the lack of references, mainly for species that are phylogenetically related, such inferences should be made with caution. Among the fucosylated proteins, the more commonly identified in human tears are lactrin, lactoferrin and mucins, all of which play an important role in the immune innate barrier [6, 7, 21]. Another characteristic of the caiman glycoproteins tear film is the presence of molecules with an enzymatic binding function. Both characteristics (fucosylation and enzymatic binding) suggest that these components may play a crucial role in the protection of these reptiles’ eyes against pathogens [17]. It is worth noting that not all glycoproteins found in the caiman tear film are linked to fucose.

In humans, N-type glycoproteins are the main tear film PTM, and O-glycans can be linked with other components, such as mucins from the corneal epithelium glycocalyx, and are possibly not dispersed in the aqueous portion of the tear film, or can be present at very low concentrations [5, 6, 12, 19]. This situation can explain the fact that O-linked glycoproteins were not found in our study. Moreover, species-specific situations must be also considered. In this way, subsequent studies using more sensitive methodologies may show the presence of these O-glycans and others PTMs types, such as lipid-linked glycans. An example of such methodology was described Ozcan et al (2013) [5], who used an alkaline borohydride solution as an enrichment step with the objective to detect O-linked glycans. There are no studies showing the interference of the collection method in the obtaining of O-glycans. In addition, it was already described that there are no significant differences in the protein composition of tears when samples collected by different methods (capillary tube and STT) were compared [18].

In human tears, the most abundant proteins and the most frequent ontological characteristics are associated with the tear’s major functions [21]. Thus, the abundance of proteins such as ovotransferrin and the uncharacterized proteins may be associated with glycoproteins’ major functions in tears, such as stabilization and immune defense. Ovotransferrin is an extracellular component with associated biological processes and molecular functions such as iron-binding and antimicrobial properties. This component is a glycoprotein found in the egg albumen and in the bird serum that is increased in inflammatory situations [26]. Similar characteristics are observed for lactoferrin, found in the human tear film, an iron binding glycoprotein with a marked activity in the innate immune response since certain bacteria are dependent on iron [12, 20, 21]. The iron binding property may be associated with environmental influences or even to an adaptation process of birds [10], and a previous study on the *R. magnirostris* tear film showed high concentrations of sodium, chlorine and iron in the tear film [16].

The most abundant caiman tear glycoprotein, uncharacterized proteins, were also presented a wide array of ontological characterizations, possibly derived from the different peptide bonds with oligosaccharides. The uncharacterized proteins were more abundant in the proteomic tear study conducted by Raposo et al 2020, and they are correlated to carbohydrate metabolic functions [17]. The broad-snouted caiman tear film unit also presents a high concentration of urea and iron. This complex fluid can allow the singular stability in the interface with freshwater, and the overall stability of the ocular surface may be an attribute of these uncharacterized proteins, as described for humans [10, 16]. Further studies are needed to characterize these proteins functions in the caiman tear film.

In the human tear film, N-type peptides are described as extracellular components with enzymatic function and secreted by the Golgi complex [3]. The ontological characterization for both the bird and caiman species showed that most of the glycoproteins are present in the extracellular space or as a component of the cell membrane. These glycoproteins have been described in the human tear film as having many different functions, such as immunological signaling [1, 27]. The diversity of biological processes reflects the information on these PTMs: different oligosaccharides may perform different functions and promote a marked diversity among eukaryotes [3]. The endopeptidase inhibition activity was the most frequent molecular function for both species studied herein, which was also described as the main function in a study based on human tear film proteomics [21]. Although this molecular function is the most frequently described in the tear film of birds, ovotransferrin possess a marked iron binding activity. While endopeptidase inhibition activity is the most frequent function found herein for hawk tear film glycoproteins, ion binding represented the second largest percentage. These two functions coincide with the large amount of ovotransferrin as well as a high abundance of glycoproteins that have endopeptidase inhibition activity, such as kininogen-1 and alpha-2-macroglobulin-like. Another possibility is that a harmonic stability between the functions of these components is crucial to maintain tear film stability, as previously described by Raposo et al [17] for these same species, when analyzing the protein composition of their tears and the different functions of these molecules.

The hawk and caiman tears had different glycocomponents when compared to human tears. The difficult acquisition of tear film samples in these species did not allow a comparison between individuals, and this situation limits the present discussion. Another limitation was the small number of hawks that were available for obtaining samples. In addition, the scarcity of information included in the hawk and caiman genetic banks did not allow a more specific characterization of these glycoproteins. A further description and understanding of these glycoproteins may reveal molecules with a biotechnological potential for use in human or animal health, mainly in the prevention of lesions during exposure to adverse conditions, as they are able to maintain the stability of the tear fluid in animals that are exposed to such events.

## Conclusion

This is the first study, to the best of our knowledge, to describe the glycoproteins present in non-mammalian tears; 51 glycoproteins were found in hawk tears and 29 in caiman tears, all of them linked to the N-type. These glycoproteins were found mainly in extracellular spaces, and their molecular function correlated with endopeptidase inhibitory activity. These characteristics reveal a similarity to those described for human tear glycoproteins, which suggests that the tear stability and maintenance mechanisms are similar. However, differences such as the high presence of uncharacterized proteins and the enzymatic function shows that perhaps these tear films have characteristics that were developed during the adaptation to different environments.

## Methods

### Ethics Approval and Consent to Participate

This study was registered at the System of Authorization and Information on Biodiversity (protocol no. 27489) and at the National System of Management of Genetic Heritage (protocol no. A1F8C27), both part of the Brazilian Ministry of the Environment, and approved by the Ethics Committee on Animal Experimentation of the School of Veterinary Medicine and Zootechnology of UFBA (protocol no. 72/2016). All procedures were conducted in compliance with the Association for Vision and Ophthalmology Research (ARVO) and the National Institutes of Health (NIH) for the use of animals in eye and ophthalmic research. All methods were performed in accordance with the relevant guidelines of ARRIVE (Animal Research: Reporting of In Vivo Experiments). In addition, at all stages involving contact with the animals, minimally invasive maneuvers were performed to reduce stress and pain.

### Animal Species Included in the Experiment

A total of 12 healthy adult hawks of unknown sex were screened in this study. All animals were kept at the Center for Triage of Wild Animals (Salvador, Brazil) and housed in an outdoor enclosure. Seventy-one healthy adult caimans, males (*n*=48) and females (*n*=23), were used in this study. These animals were kept in a commercial breeding center (Alagoas State, Brazil). These two wild animal species were chosen because they are from two different ecological niches, and both were readily available for sampling and clinical evaluation.

### Tear Collection and Sample Preparation

All collection procedures were conducted under physical restraint without the use of anesthetics and sedatives. The animals inhabited enclosures like the natural habitat, and in this way, they presented some dirt in the periocular region that could contaminate the samples, such as dust, indoor enclosure substrates and excretes. Because of this situation, the periocular region of all animals was previously cleaned using a cotton moistened with sterile water. At the time of cleaning, the handler gently kept the animals' eyelids closed, such that the fluid did not reach the ocular surface. The cleaning did not reach the ocular surface. In this moment, trained veterinary ophthalmologists performed a routine physical examination, based on the examination of the eyes and periocular regions using light and a magnifying glass set at a 4x magnification (Lupa Pala, Optivisor, São Paulo, Brazil), avoiding proximity to the animal with the objective to reduce stress. This step was made to ensure that the animals did not have alterations in the evaluated region and that the samples were not contaminated by water. After 20 minutes, the collection was made according to Cardoso-Brito and collaborates (2018) [28]. Other ophthalmic evaluations, such as biomicroscopy (SL-17, Kowa Medical, Torrance, CA) and the fluorescein test (Ophthalmos, São Paulo, Brazil) were performed after the acquisition of the samples since these techniques can influence tear production as a consequence of the proximity to the animals and an extended handling time (these are well known stress inducers). If diseases or morphological changes were found, the sample was excluded from the evaluation.

All collections were performed in the morning (08;00–11;30 h) and the procedures were made according to previous similar studies [10, 16, 17]. Tear collection from all animals was made using a Schirmer strip (Tear Flo Test Strips, Oasis, San Dimas, CA) inserted into the lower conjunctival sac with the objective to absorb basal and reflex tears. The strip was placed according to the test instructions, with the chamfered region (5 mm) inserted in the conjunctival sac, similar to what was performed in birds and reptiles in an ophthalmic tear evaluation semiotechnique [15, 29]. The eyelids remained naturally open and only one strip was inserted into each eye to reduce the exfoliative effect on conjunctival cells [30]. Similar to Raposo and collaborators [10], strips were maintained in the fornix until the moistened portion reached 30mm according to test instructions. If the strip felt or the animal did not allow manipulation, the collection on the specific animal was stopped. The strips were then centrifuged, and samples were pooled for each species and frozen at -80°C until processing.

Tear proteins were diluted with ammonium bicarbonate, reduced with 18 mM dithiothreitol, alkylated with 27 mM iodoacetamide, and incubated overnight with 1 μg trypsin at 37 °C. The resulting glycopeptides were enriched by solid-phase extraction using an iSPE-HILIC cartridge (The Nest Group, Boston, MA). Cartridges were conditioned with acetonitrile and 0.1% (v/v) trifluoroacetic acid (TFA) in water, followed by 1% TFA and 80% acetonitrile in water. Peptides were loaded onto the column and washed with 1% TFA and 80% acetonitrile in water. Enriched products were eluted with a solution of 0.1% TFA in water and dried [23, 30].

Samples were loaded using 2% (v/v) acetonitrile and 0.1% (v/v) TFA in water and separated using a reverse-phase Michrom Magic C18AQ column (200 μm, 150 mm) coupled with a Q Exactive Plus mass spectrometer through a Proxeon nano-spray source (Thermo Scientific, Waltham, MA). A binary gradient was applied at a 2 μL/min constant flow rate using 0.1% (v/v) formic acid in (A) water and (B) 100% acetonitrile. For acquisition, the instrument was run in a data-dependent mode as follows: spray voltage, 2.2 kV; ion transfer capillary temperature, 200°C; full scan mass range, m/z 350-1600; MS automatic gain control, 1 x 106; MS maximum injection time 30 ms; MS/MS automatic gain control, 5 x 104; MS/MS maximum injection time 50 ms; dynamic exclusion 10 s; precursor resolution, 70000; product ion resolution, 17500 ; precursor ion isolation width of m/z 1.6; stepped collision energy higher-energy collisional dissociation [23, 31].

Raw data were exported using xCalibur, version 2.0 (Thermo Scientific). Proteins were identified from the tandem mass spectra using Byonic version 2.7.4 (Protein Metrics, Cupertino, CA); polypeptides with a score >log 2 were included, and those below this score were considered foreign proteins. The data were evaluated using UPKB (the central hub for the collection of functional information on proteins [32]), with the research criterion set to the taxa Aves (class) and Alligatoridae (family) for hawk and caiman, respectively. These research criteria were chosen due to the abundance of matches found. The amount of match per species was small or non-existent because of the fact that proteins from these species have not been previously described. The UPKB consists of two sections: Reviewed (Swiss-Prot), with records based on information extracted from the scientific literature and curator-evaluated computational analysis, and Unreviewed (TrEMBL), with records that await full manual annotation. Both sections were used in this study. The parameters were set to mass tolerance of 5–10 ppm for the precursor and 10–20 ppm for fragment ions; peptide probability >0.95; carbamidomethylation of cysteine as a fixed modification; oxidation of methionine and tryptophan, deamidation of asparagine and glutamine, acetylation of the protein N terminus, and ammonia loss of cysteine as variable modifications; *N*-linked glycosylation of asparagine (309 entries); two missed cleavage sites. Identifications were filtered with a 1% false discovery rate and were accepted if the following conditions were met: |Log Prob| > 2; Delta Mod > 10.

The results were organized into clusters, based on the frequency of the protein and its ontological category (cellular components, biological processes and molecular function, according to UPKB) as previously described in studies on tear humans [20–22] and reproduced for animal samples [17]. The top scoring clusters were summed up and the percentage contribution of each annotation cluster was determined and represented in the form of a pie-chart. The pie charts comprised of the annotation clusters represented by the name of the one of its members that seemed most biologically relevant.

## Supplementary Information

**Additional file 1.**

**Additional file 2.**

## Data Availability

The datasets used and/or analyzed during the current study are available from the corresponding author on reasonable request.

## Abbreviations

PTMs: Post-translational modifications
MS: Mass spectrometry
LC-MS: Liquid chromatography–mass spectrometry
UPKB: UniProt Knowledgebase
TFA: Trifluroacetic acid
