# Supplementary material for: The glycoproteomics of hawk and caiman tears
Source: BMC Vet Res. 2021 Dec 9;17:381. doi: 10.1186/s12917-021-03088-1 (PMC8656020; doi:10.1186/s12917-021-03088-1)
Supplement: Supplementary file 1 — Additional file 1. [file 12917_2021_3088_MOESM1_ESM.docx]

**Table 1.** Glycoproteins and glycosylated peptides identified in the roadside hawk tear (*Rupornis magnirostris)*.

| no. | ID | Protein name | m/z | Score | Formerly glycosylated peptide | Glycans |
| --- | --- | --- | --- | --- | --- | --- |
| 1 | A0A093FHI9 | Serum albumin | 1127.029 | 677.90 | K.QSDIETC[+57.021]FGEEGANLIVQSR.A | - |
| 2 | A0A091U600 | Tetranectin (Fragment) | 1172.859 | 687.80 | K.AMIDN[+1971.719]ISQEVALLK.E | HexNAc(5)Hex(5)Fuc(1)NeuAc(0) |
| 3 | A0A091NPM5 | Serum albumin (Fragment) | 748.430 | 676.50 | K.AVTLITFAQYLQK.C | - |
| 4 | A0A0Q3US23 | Kininogen-1 | 1099.782 | 1101.80 | K.IFPQVN[+1913.677]C[+57.021]SK.E | HexNAc(4)Hex(5)Fuc(0)NeuAc(2) |
| 5 | A0A0A0A1J2 | Alpha-2-macroglobulin (Fragment) | 1030.436 | 497.00 | R.N[+1784.634]GSNMVIVDVK.M | HexNAc(4)Hex(5)Fuc(0)NeuAc(1) |
| 6 | A0A093GN98 | Angiotensinogen (Fragment) | 1448.605 | 1041.80 | R.VYVHPFNLFSFN[+2075.730]K.S | HexNAc(5)Hex(7)Fuc(0)NeuAc(2) |
| 7 | A0A091T667 | Ovostatin (Fragment) | 1886.779 | 502.70 | R.SVAIQNVDNTVFIQTDKPIYKPGQK.V | HexNAc(2)Hex(9)Fuc(0)NeuAc(0) |
| 8 | A0A0Q3PLB4 | Antithrombin-III | 1097.795 | 572.60 | R.KIINEWVAN[+2204.772]K.T | HexNAc(4)Hex(5)Fuc(0)NeuAc(2) |
| 9 | A0A091V8N3 | Alpha-1-antiproteinase 2 (Fragment) | 1141.991 | 476.50 | K.GLAFN[+2204.772]LTEIEEQEIHEGFQR.L | HexNAc(4)Hex(5)Fuc(0)NeuAc(2) |
| 10 | Q5ZMQ2 | Actin cytoplasmic 2 | 1275.599 | 793.70 | K.LC[+57.021]YVALDFEQEMATAASSSSLEK.S | - |
| 11 | A0A0A0ADU8 | Alpha-2-macroglobulin-like 1 (Fragment) | 955.9264 | 520.60 | R.ATSLGEVN[+1930.692]ITVSTEALSSK.E | HexNAc(4)Hex(6)Fuc(1)NeuAc(0) |
| 12 | U3K7W5 | Lumican | 1385.953 | 596.00 | K.LHINYNN[+2133.772]LTEAVGPLPK.T | HexNAc(5)Hex(5)Fuc(1)NeuAc(1) |
| 13 | A0A093FBG5 | Ig heavy chain V-III region VH26 (Fragment) | 847.412 | 619.50 | R.DNSQSTVTLQMNSLR.A | - |
| 14 | A0A091QSZ2 | Vitamin D-binding protein | 803.044 | 579.70 | R.TFLSM[+15.995]VSTC[+57.021]C[+57.021]ISPAPTAC[+57.021]FLK.E | - |
| 15 | A0A091HBG2 | Ovotransferrin (Fragment) | 1233.243 | 429.90 | K.EFLGDQYYASVASLNTC[+57.021]NPSDLLQVC[+57.021]TFLEDK | - |
| 16 | A0A091JW70 | Vitamin D-binding protein (Fragment) | 812.755 | 830.60 | R.FLHEYASSYSQAPLPVLLGSTR.S | - |
| 17 | A0A093GS00 | Alpha-1-antiproteinase 2 (Fragment) | 1586.157 | 472.80 | R.DTN[+2075.730]STLFIGK.I | HexNAc(4)Hex(6)Fuc(0)NeuAc(1) |
| 18 | A0A099ZM43 | Serum albumin | 738.423 | 798.90 | R.VSLLGHFIYTVAR.R | - |
| 19 | A0A091G4B1 | Coagulation factor IX (Fragment) | 1186.5228 | 696.40 | K.ILPHPTYN[+2075.730]ATINK.H | HexNAc(4)Hex(6)Fuc(0)NeuAc(1) |
| 20 | A0A091HI50 | Glyceraldehyde-3-phosphate dehydrogenase (Fragment) | 794.449 | 449.40 | K.LVINGNAITIFQER.D | - |
| 21 | A0A0A0AN62 | Serum albumin | 774.405 | 720.20 | K.M[+15.995]PQVSTDTLLEIGK.K | - |
| 22 | A0A093BJI5 | Complement C3 (Fragment) | 834.965 | 465.20 | R.PYTVALTSYALALTGK.L | - |
| 23 | A0A093CFV7 | Ig heavy chain V-III region CAM (Fragment) | 847.906 | 273.80 | R.DDSQSTVTLQMNSLR.A | - |
| 24 | A0A093Q8F2 | Protein S100-A4 | 979.499 | 339.40 | M.[+42.011]AC[+57.021]PLEQALAVMVTTFHK.Y | - |
| 25 | A0A093KM83 | Ovotransferrin (Fragment) | 935.480 | 546.30 | R.IPPLMDSQLYLGFEYYSAIQSLQK.D | - |
| 26 | U3K0R0 | Albumin | 976.920 | 419.40 | K.TDNPAEC[+57.021]YGNAQEELNK.Q | - |
| 27 | S5N721 | Complement component 3d (Fragment) | 1032.212 | 587.40 | R.TYNIEGTSYALLALLQM[+15.995]EKPELTGPVAR.W | - |
| 28 | A0A0Q3MG37 | Multiple inositol polyphosphate phosphatase 1 | 1315.864 | 228.30 | K.N[+2075.730]VTSPWC[+57.021]SLFSEEDAK.V | HexNAc(4)Hex(6)Fuc(0)NeuAc(1) |
| 29 | A0A091W4S3 | Myeloperoxidase (Fragment) | 839.740 | 546.60 | R.EQINAVSSFIDASTVYGSEDSVAK.S | - |
| 30 | A0A091J8H4 | Lysozyme g (Fragment) | 1070.866 | 565.10 | R.YGNILNVDTTGASEATAKPEGLSYAGVPASEK.I | - |
| 31 | A0A091URA2 | Deleted in malignant brain tumors 1 protein (Fragment) | 817.619 | 375.60 | R.VEVLHEEQWGSVC[+57.021]HDDWDLNDAQVVC[+57.021]K.Q | - |
| 32 | A0A091P6E7 | Inter-alpha-trypsin inhibitor heavy chain H2 (Fragment) | 1178.902 | 407.70 | K.IQFNYPQESVSDVTQSSFHNYFGGSEIVVAGK.V | - |
| 33 | A0A091UTV5 | Ig lambda-1 chain C regions (Fragment) | 702.361 | 305.20 | R.TLSSGIETSQPQR.Q | - |
| 34 | A0A091VQC7 | Lysozyme g (Fragment) | 858.420 | 529.50 | K.IVGSWDSEEHLAQGTEILC[+57.021]GMIK.E | - |
| 35 | A0A091HVD5 | Gelsolin (Fragment) | 914.453 | 363.80 | R.GAAAIFTVQMDDYLQGK.A | - |
| 36 | A0A091UMI1 | Beta-2-glycoprotein 1 (Fragment) | 1506.968 | 312.00 | K.SC[+57.021]AYTVAVQC[+57.021]VDGN[+2043.740]LTLPAC[+57.021]FK | HexNAc(4)Hex(4)Fuc(2)NeuAc(1) |
| 37 | A0A099ZXZ8 | Ovoinhibitor (Fragment) | 850.364 | 286.40 | R.QETSEIDC[+57.021]SQYPSR.M | - |
| 38 | A0A0A0B2C1 | Deleted in malignant brain tumors 1 protein (Fragment) | 1128.509 | 271.60 | R.LGTVC[+57.021]DDFWDLSDAQVVC[+57.021]R.Q | - |
| 39 | A0A091U489 | Prothrombin (Fragment) | 1262.484 | 236.90 | R.NPDN[+2075.730]NSEGPWC[+57.021]YTR.D | HexNAc(4)Hex(6)Fuc(0)NeuAc(1) |
| 40 | A0A091W0N8 | Transketolase | 783.9038 | 413.90 | R.TVAFASTFATFFTR.A | - |
| 41 | A0A091URM5 | Protein S100 (Fragment) | 866.936 | 213.20 | R.EFLAFMNTELAAFTK.N | - |
| 42 | A0A0A0AG24 | Vitronectin (Fragment) | 1400.567 | 384.40 | K.N[+1872.651]GSLYAFR.G | HexNAc(3)Hex(6)Fuc(0)NeuAc(1) |
| 43 | A0A091UX80 | Rab GDP dissociation inhibitor (Fragment) | 854.937 | 173.10 | K.YIAIASTTVETADPEK.E | - |
| 44 | Q5ZKC9 | 14-3-3 protein zeta | 1028.010 | 241.20 | K.GIVEQSQQAYQEAFEISK.K | - |
| 45 | A0A091WLT5 | Aminopeptidase N (Fragment) | 1271.541 | 262.20 | K.AN[+2204.772]FTVTLIHPSDHR.A | HexNAc(4)Hex(5)Fuc(0)NeuAc(2) |
| 46 | A0A093BW72 | Anterior gradient protein 2 | 777.925 | 206.90 | K.FVLLNLVYETTDK.N | - |
| 47 | A0A091TNQ5 | Complement component C9 (Fragment) | 1051.777 | 242.70 | K.N[+2204.772]SSLSSILK.S | HexNAc(4)Hex(5)Fuc(0)NeuAc(2) |
| 48 | A0A093N8L5 | Plasminogen (Fragment) | 1145.107 | 275.60 | K.MPWC[+57.021]YTTN[+2204.772]R.T | HexNAc(4)Hex(5)Fuc(0)NeuAc(2) |
| 49 | A0A091GLR6 | Fibronectin (Fragment) | 1356.235 | 316.50 | R.DQC[+57.021]IVDGITYDVN[+1913.677]QTFHK.R | HexNAc(4)Hex(5)Fuc(0)NeuAc(1) |
| 50 | A0A093NJT8 | Ovostatin (Fragment) | 896.453 | 160.70 | K.EYQFFTVEEYVLPK.F | - |
| 51 | A0A091PHV6 | Alpha-1-antichymotrypsin (Fragment) | 1060.457 | 171.50 | K.IINEWVAN[+2092.745]K.T | HexNAc(4)Hex(7)Fuc(1)NeuAc(0) |

- no data available

no: number order; ID: identification according to UniProt Knowledgebase; m/z: mass-to-charge ratio; score: score from liquid chromatography–mass spectrometry
