# Supplementary material for: The glycoproteomics of hawk and caiman tears
Source: BMC Vet Res. 2021 Dec 9;17:381. doi: 10.1186/s12917-021-03088-1 (PMC8656020; doi:10.1186/s12917-021-03088-1)
Supplement: Supplementary file 2 — Additional file 2. [file 12917_2021_3088_MOESM2_ESM.docx]

**Table 2.** Glycoproteins and glycosylated peptides identified in the caiman tear (*Caiman latirostris)*.

| no. | ID | Protein name | m/z | Score | Formerly glycosylated peptide | Glycans |
| --- | --- | --- | --- | --- | --- | --- |
| 1 | A0A151NFL2 | Uncharacterized protein | 1268.564 | 472.00 | K.FSQEEAVPASN[+1971.719]VSLLIK.A | HexNAc(5)Hex(5)Fuc(1)NeuAc(0) |
| 2 | A0A151NL74 | Uncharacterized protein | 1206.505 | 819.20 | K.FQDSPLQN[+1702.581]FSTQFEVK.E | HexNAc(2)Hex(8)Fuc(0)NeuAc(0) |
| 3 | A0A1U8DSS5 | Olfactomedin-4 | 800.450 | 422.50 | K.VEQLEIIAQTLSQK.F | - |
| 4 | A0A151MC38 | Proteasome subunit alpha type | 1229.200 | 60.00 | K.N[+2799.036]VSIGIVGK.D | HexNAc(7)Hex(4)Fuc(3)NeuAc(1) |
| 5 | A0A1U8DKB4 | Actin. cytoplasmic 2 isoform | 939.431 | 602.60 | M.[+42.011]EEEIAALVIDNGSGMC[+57.021]K.A | - |
| 6 | A0A151LZ07 | Histone | 846.949 | 717.50 | R.HLQLAIRNDEELNK.L | - |
| 7 | A0A151MYS3 | Uncharacterized protein | 988.072 | 266.20 | R.N[+1913.677]STVIC[+57.021]QVK.D | HexNAc(4)Hex(5)Fuc(0)NeuAc(1) |
| 8 | A0A151MT59 | Uncharacterized protein | 1154.505 | 543.30 | K.NANLAN[+2133.772]LTQQLK.T | HexNAc(5)Hex(6)Fuc(1)NeuAc(0) |
| 9 | A0A151NLJ0 | Uncharacterized protein | 1534.639 | 135.00 | R.LAN[+2279.830]ETLK.V | HexNAc(5)Hex(6)Fuc(2)NeuAc(0) |
| 10 | A0A151MF96 | Uncharacterized protein | 1295.494 | 369.40 | R.FN[+2772.984]ESC[+57.021]IVDK.K | HexNAc(6)Hex(6)Fuc(0)NeuAc(2) |
| 11 | A0A151N583 | Uncharacterized protein | 709.349 | 586.50 | K.VVVDLAHTC[+57.021]VANVDAPGC[+57.021]TK.S | - |
| 12 | A0A151N542 | Calumenin isoform C | 961.793 | 82.70 | K.N[+3706.353]ATYGYILGK.I | HexNAc(7)Hex(6)Fuc(5)NeuAc(2) |
| 13 | A0A1U7S8S7 | Fumarylacetoacetate hydrolase domain-containing protein | 1351.906 | 75.00 | K.N[+3050.125]GTQWVLGK.S | HexNAc(6)Hex(5)Fuc(5)NeuAc(1) |
| 14 | A0A151MNZ5 | Desmocollin-2 isoform B | 846.703 | 144.70 | R.AN[+1751.624]VTLLR.I | HexNAc(4)Hex(4)Fuc(0)NeuAc(1) |
| 15 | A0A151N5S7 | Serum albumin | 762.426 | 548.30 | K.EVALITFAQYLQK.C | - |
| 16 | A0A151NNS5 | Phosphoglycerate kinase | 877.891 | 377.60 | K.GC[+57.021]ITIIGGGDTATC[+57.021]C[+57.021]AK.W | - |
| 17 | A0A151PJ40 | Tropomyosin alpha-3 chain isoform | 1190.473 | 135.80 | K.N[+3956.422]VTNNLK.S | HexNAc(6)Hex(7)Fuc(5)NeuAc(3) |
| 18 | A0A1U8E129 | Alpha-2-macroglobulin-like protein 1 | 985.075 | 318.40 | R.N[+2491.946]VTK.S | HexNAc(7)Hex(3)Fuc(4)NeuAc(0) |
| 19 | A0A1U8D0F8 | heterogeneous nuclear ribonucleoprotein K isoform X4 | 863.796 | 429.40 | R.IITITGTQDQIQNAQYLLQNSVK.Q | - |
| 20 | A0A1U7SGY2 | Alpha-enolase | 1058.955 | 268.50 | K.AVAHVN[+1378.476]K.T | HexNAc(2)Hex(6)Fuc(0)NeuAc(0) |
| 21 | A0A151NFJ9 | Uncharacterized protein | 1182.501 | 423.60 | R.TFDIAIN[+2116.756]VSYTGK.R | HexNAc(5)Hex(5)Fuc(0)NeuAc(1) |
| 22 | A0A1U7SXA4 | Uncharacterized protein | 889.350 | 232.70 | K.N[+2204.772]LSK.I | HexNAc(4)Hex(5)Fuc(0)NeuAc(2) |
| 23 | A0A151MN05 | Tumor protein D52 isoform A | 829.449 | 282.40 | K.VEEEIQTLSQVLAAK.E | - |
| 24 | A0A1U7RUW6 | Protein-glutamine gamma-glutamyltransferase | 715.377 | 335.80 | K.VNIIAEVQETGEK.V | - |
| 25 | A0A151MZ29 | Prestin isoform C | 1396.911 | 78.20 | R.N[+3374.231]ATQLLR.F | HexNAc(6)Hex(7)Fuc(5)NeuAc(1) |
| 26 | A0A1U7RTA3 | Alpha-1-antitrypsin-like | 1144.212 | 302.80 | K.SLYEAESSSSNFQN[+2408.872]SSEAVK.E | HexNAc(5)Hex(5)Fuc(2)NeuAc(1) |
| 27 | A0A1U8DQE8 | ATPase family AAA domain-containing protein 5 | 1154.141 | 232.70 | K.N[+2759.030]ITPEK.C | HexNAc(6)Hex(5)Fuc(5)NeuAc(0) |
| 28 | A0A151MYW1 | Ig epsilon chain C region | 1155.467 | 326.00 | R.AN[+2059.735]VTEEEWIEGK.T | HexNAc(4)Hex(5)Fuc(1)NeuAc(1) |
| 29 | A0A151NLJ0 | Uncharacterized protein | 1244.757 | 108.70 | R.N[+4159.501]LSVEVR.S | HexNAc(7)Hex(7)Fuc(5)NeuAc(3) |

- no data available

no: number order; ID: identification according to UniProt Knowledgebase; m/z: mass-to-charge ratio; score: score from liquid chromatography–mass spectrometry
